# Role of telomere length and telomerase activity in accelerated cellular aging and major depressive disorder: a systematic review

**DOI:** 10.1038/s41380-025-03296-3

**Published:** 2025-10-07

**Authors:** Samantha Wai Sam Au Young, Chuin Hau Teo, Ishwar S. Parhar, Tomoko Soga

**Affiliations:** 1 Jeffrey Cheah School of Medicine and Health Sciences, Monash University Malaysia, Jalan Lagoon Selatan, 47500 Bandar Sunway, Selangor Malaysia; 2 Centre Initiative for Training International Researcher, University of Toyama, Gofuku, Toyama, Japan

**Keywords:** Diagnostic markers, Neuroscience

## Abstract

Recent research has increasingly focused on understanding the relationship between cellular aging and mental health, particularly Major Depressive Disorder (MDD). Telomeres, protective structures at the end of chromosomes, and telomerase, an enzyme responsible for their maintenance, have emerged as potential markers of cellular aging and targets for therapeutic interventions in MDD. This review synthesizes findings from 30 studies conducted over the past 15 years, examining alterations in telomere length (TL) and telomerase activity (TA) in individuals with MDD compared to healthy controls. Most studies reported shorter TL in MDD patients, particularly in cases of chronic or severe depression, determined by the duration of illness or illness episode and by measurements of depression severity (e.g. HAM-D, BDI, etc.), suggesting an association between MDD and accelerated cellular aging. Elevated TA was also observed in MDD, with potential implications for treatment response. However, conflicting findings and methodological variations highlight the complexity of the relationship between TL, TA, and MDD, warranting further research. Additionally, studies investigating other biomarkers of cellular aging, such as mitochondrial DNA, provide further insights into the pathophysiology of MDD. Studies on brain cells reveal regional variations in telomere dynamics, suggesting a nuanced relationship between depression and cellular aging across different brain regions. While evidence suggests a potential reversibility of TL alterations in MDD, further research is needed to elucidate underlying mechanisms and develop targeted interventions. Overall, this review underscores the importance of understanding cellular aging processes in MDD and their potential implications for diagnosis, treatment, and the development of novel therapeutic strategies.

## Introduction

Major depressive disorder (MDD) has contributed significantly to the global burden of mental disorders in recent years. With an estimated 3 million people being affected worldwide, these cases of MDD are projected to increase exponentially as a result of the recent COVID-19 outbreak [1]. Besides mental illness, individuals with MDD have an average life expectancy that is approximately ten years shorter than the healthy population [2]. This is a significant difference, and it is important to understand the biomedical mechanisms of this disparity. Therefore, it has been hypothesized that MDD is associated with accelerated age-related biological and functional decline [3, 4]. Recent research has seen a shift towards understanding the intricate relationship between cellular biology and mental health, particularly MDD. This change moves beyond the traditional views of telomeres and telomerase solely in cellular aging to explore their potential roles in mental health. One potential biomarker under study for a possible relationship between MDD and biological aging is leukocyte telomeres. Leukocyte telomeres are quantified by their length and the activity of telomerase.

Telomeres are repetitive DNA sequences located at the ends of eukaryotic chromosomes, playing a vital role in maintaining genomic stability and integrity by safeguarding against chromosomal aberrations. Chromosomal structures are known to be crucially involved in the process of cellular ageing. Telomerase, an enzyme responsible for elongating and maintaining telomeres, counteracts the natural attrition that occurs during cellular division, thus preserving chromosomal integrity. Traditionally, the shortening of telomeres has been associated with the aging process and age-related diseases, often attributed to prolonged exposure to inflammation, high levels of cortisol, and oxidative stress [5, 6]. Therefore, Telomerase Length (TL) and telomerase activity (TA) are used as biomarkers of both aging and aging-associated pathological conditions. In 2006, Simon et al. assessed leukocyte telomere lengths (LTLs) in MDD individuals, finding them significantly shorter compared to controls, indicating an accelerated aging process. Since then, studies have shown a complex relationship between telomere shortening and MDD, with limited research on LTLs and its potential restorative response to antidepressants [5, 7, 8]. While various animal models have investigated the effects of antidepressants on telomere biology, limited research exists on the connection between LTLs, TA and antidepressant response in MDD patients [7–10].

There are a limited number of references that investigate neuronal aging in the brain of patients with MDD. However, TL and TA in the limbic area, especially the hippocampus, has been studied and linked with accelerated aging in patients with MDD. TL shortening and alteration of TA could represent a mechanism that moderates the proliferative capacity of human hippocampal progenitors, which may subsequently impact human cognitive function and mental health pathophysiology. Some studies investigate potential correlations between peripheral markers of cellular aging and their implications for brain cell function in MDD, including examining cellular senescence reversibility and its role in hippocampal neurogenesis and antidepressant treatments [11]. The significance of decreased TL in the hippocampus of individuals with MDD is highlighted by its association with depressive disorder pathophysiology [11], hippocampal volume reductions in MDD compared to controls, and neurogenesis [12]. Studies show an association between shorter leukocyte telomeres and reduced hippocampal volume [13].

Previous reviews (2006–2015) noted decreased LTLs in full syndrome MDD, especially in chronic or severe depression, though with a small effect size. Building upon this foundation, this review aims to update this analysis from 2015 onwards, exploring emerging changes and research gaps. Fundamentally, we aim to illuminate the complex interplay between molecular mechanisms and psychiatric pathology, providing insight into telomeres and telomerase roles in mental health disorders. Additionally, the review discusses other biomarkers like human telomerase reverse transcriptase (hTERT) mRNA expression and cell-free mitochondrial DNA (cf-mtDNA) in MDD neurobiology and their relevance in telomere dynamics and cellular aging [9, 14–19].

We selected studies from 2009 onwards and focused on studies involving human subjects aged 19–65 diagnosed with unipolar MDD, emphasising clinical investigations on the relationship between MDD and changes in TL and TA, and other relevant biomarkers of cellular senescence. The main body of the review is divided into several sections, beginning with the results and discussion, presenting the main findings regarding the relationship between MDD and cellular aging, specifically focusing on TL and TA. Subsequent sections cover additional subtopics that have emerged over the past decade, including the association of MDD and cellular aging in the brain, other biomarkers of cellular aging in MDD, and the reversibility of cellular aging.

## Methods

This literature review utilized PubMed and OVID databases, employing keywords such as ‘telomeres,’ ‘telomerase,’ ‘major depressive disorder,’ and ‘depression’ to identify relevant studies over the past 15 years. We selected studies from 2009 onwards and focused on studies involving human subjects aged 19–65 diagnosed with unipolar MDD, emphasising clinical investigations on the relationship between MDD and changes in TL and TA, and other relevant biomarkers of cellular senescence. The identified articles were thoroughly reviewed. This systematic review followed the PRISMA 2020 flow diagram for a set of reporting standards.

## Results

**Fig. 1:**
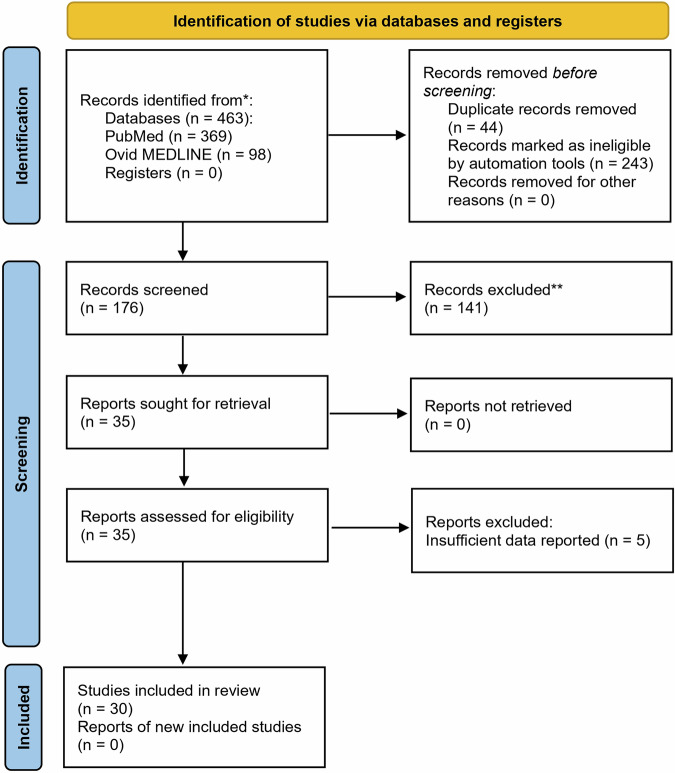
Prisma Flow Diagram Screening for Research Articles Examining Telomere Length and Telomerase Activity in Major Depressive Disorders.

This review provides a comprehensive overview (Fig. 1) and critical analysis of studies examining TL and TA in MDD, as outlined in Tables 1, 2 and 3. A total of thirty studies focusing on TL and/or TA were reviewed. Overall, eighteen of the twenty-five TL studies showed decreased telomere length associated with MDD, and all five of the TA studies demonstrated higher TA associated with MDD. Three studies examining human brain tissue in Table 3 shed light on the intricate relationship between MDD and TL and/or TA dynamics.

**Table 1:** Summary of Telomere Length in recent Major Depressive Disorder clinical studies.

Author, Year	Sample size: MDD / controls	Female (%): MDD/ controls	Duration of study	Attrition rate	Main findings: TL in MDD	Other findings
Ochi, 2023 [10] 10.1038/s41380-023-02263-0	33 / 20	78.8% / 40%	–	–	LTL was significantly reduced in adolescents with MDD. Significant correlation found between depression severity, comorbid anxiety (especially suicidality) and TL.	mtDNAcn significantly increased in MDD adolescents.
da Silva, 2022 [5] 10.1097/ypg.0000000000000305	12 / 12	92%	24 weeks	–	Shorter TL in MDD group (p < 0.0001)	–
Huang, 2022 [16] 10.1080/15622975.2021.2013091	70 / 51	60% / 68.6%	–	–	Shorter TL in MDD group (p = 0.042)	–
Kuehl, 2022 [23] 10.1016/j.psyneuen.2022.105762	MDD+ / ACE+: 23 MDD+ / ACE-: 24 MDD- / ACE +: 22 MDD- / ACE-: 21	MDD+ / ACE+: 61% MDD+ / ACE-: 71% MDD- / ACE+: 68% MDD- / ACE-: 57%	–	–	In the adjusted model, TL was shorter in males and negatively associated with age. No significant effect of group on TL, in the unadjusted model.	There are no significant associations between the severity of MDD or ACE and TL.
Pisanu, 2020 [3] 10.1002/ddr.21612	47/ 54	70% / 56%	–	–	Shorter TL in MDD group (t = 2.30, p = 0.024)	–
Al Ahwal, 2019 [17] 10.1016/j.ajp.2018.04.039'	50	52%	–	–	TL progressively shortened from no depressive disorder to minor depression to dysthymia to major depressive disorder. TL was significantly inversely correlated with severity of depressive symptoms.	–
Vance, 2018 [26] 10.1016/j.psyneuen.2018.02.015	50 / 67	52% / 58%	2 years	n = 50 out of 166 MDD n = 67 out of 166 age- and sex-matched controls	No significant difference in baseline TL. Greater relative leukocyte TL shortening over two years (p = 0.03) in subjects with a baseline MDD. Overall correlation between relative leukocyte TL at baseline and follow-up was low (r = 0.14)	–
Verhoeven, 2018 [11] 10.1038/mp.2017.48	977 cases with depressive symptoms (no controls)	65.4%	10 years	Y15, n = 3672 (71.8%) Y20, n = 3547 (69.3%) Y25, n = 3499 (68.4%) Final n used = 977 (across 10 years with complete data)	Shorter TL in participants with higher depressive symptoms over 10 years: grand-centered (P = 0.016) CES-D means were negatively related to LTL over the 10 years. Evidence of between-person associations of depressive severity with LTL over 10 years, but not within-person.	LTL (P < 0.001) and mtDNAcn (P < 0.001) were negatively related to age. LTL was shorter across Y15, Y20 and Y25 for men (P = 0.065, P = 0.015, P = 0.019, respectively) and white individuals (P = 0.021, P o 0.001, P = 0.024, respectively.)
Whisman, 2017 [24] 10.1097/psy.0000000000000383	3609 cases with depressive symptoms (no controls)	56.3%	–	–	Shorter salivary telomeres with higher levels of depressive symptoms, in men only. Adjusted for demographics, cigarette smoking, BMI, chronic health conditions, childhood and lifetime exposure to traumatic life events, and neuroticism	–
Révész, 2016 [25] 10.1017/s0033291716000891	1569 / 608	67.5% / 61.8%	–	–	Short LTL was associated with higher symptom severity and current psychiatric diagnosis.	–
Verhoeven, 2014 [18] 10.1038/mp.2013.151	1095 (current MDD) + 802 (remitted MDD) Controls: 510	Current MDD: 67% Remitted MDD: 70% Controls: 60%	–	–	Shorter in remitted (p = 0.014) and current MDD (p = 0.012) Shorter TL was associated with higher current depression severity (p = 0.004) longer symptom duration within the past 4 years (p = 0.010)	–
Karabatsiakis, 2014 [19] 10.1186/1471-244x-14-192	Lifetime depressed with irrelevant symptoms (IS): 24 Lifetime depressed with relevant symptoms (RS): 20 Healthy controls: 50	100% women	–	–	Shorter TL (in CD4 + , CD8 + , and CD20+ cells) in unipolar MDD (with/ without clinically relevant symptoms)	–
Garcia-Rizo, 2013 [22] 10.1016/j.bbi.2012.11.009	9 / 48	Unknown gender distribution, reported as “similar”	–	–	Lower Telomere content in MDD compared to controls	–
Hoen, 2011 [21] 10.1097/PSY.0b013e31821b1f6e	206 / 746	31% / 15%	5 years	Original participants n = 952 Follow-up at 5 years n = 608	Significantly shorter LTL in MDD, controlling for age and sex; trend after controlling for additional covariates	–
Hartmann, 2010 [20] 10.1002/da.20749	54 / 20	61% / 45%	–	–	Shorter LTL in MDD	–
Ryan, 2020 [28] 10.1017/s0033291719002228	100 / 80	62%/ 67.5%	–	–	No significant difference in TL between MDD and controls.	TL itself was not associated with mood ratings and did not predict the therapeutic response to ECT. Shorter baseline TL is not a predictor of cognitive side-effects post-ECT.
Wignall, 2017 [31] 10.4088/JCP.15m10570	2710	–	–	–	Statistically significant association between depressive symptoms and TL among non-Hispanic white participants, however, not among whole sample	Telomere length was significantly associated with both age and sex.
Needham, 2015 [32] 10.1038/mp.2014.89	198 / 966	58.6% / 56%	–	–	No association between MDD and TL among those not using antidepressants. Among respondents currently taking an antidepressant, those with MD had shorter telomeres than those without.	Neither depressive nor anxiety disorders were directly associated with TL in young adults. Pharmacologically treated MDD is associated with shorter TL, likely reflecting the more severe nature of MDD that has come to clinical attention. Less severe forms of psychopathology were not associated with TL.
Simon, 2015 [6] 10.1016/j.psyneuen.2015.04.004	166 / 166	54%/ 54%	–		No significant difference in TL between MDD and controls. Higher TA in MDD overall but not statistically significant. Significantly higher TA in men with MDD even after adjustment for potential confounders.	Depression severity failed to show a significant association with both TL and TA.
Hoen, 2013 [29] 10.1017/S0033291712001766	97 / 980	64% / 53%	2 years	Total population cohort n = 1094 Follow-up population after 2 years n = 974	No association was found between depressive disorders and shorter telomeres at follow-up (baseline not reported).	Anxiety disorders predicted shorter TL at follow-up in a general population cohort.
Shaffer, 2012 [30] 10.1371/journal.pone.0048318	2225	49.9%	–	–	No statistical significance in the adjusted model	Did not include a measure of the chronicity or lifetime duration of depressive symptoms
Wolkowitz, 2011 [27] 10.1371/journal.pone.0017837	18 / 17	67% / 65%	–	–	Lifetime MDD, but not current MDD, associated with shorter TL. TL was significantly inversely correlated with lifetime depression exposure, even after controlling for age.	TL was inversely correlated with oxidative stress in both cases and controls, and with inflammation in the MDD cases.

*ACE* adverse childhood experiences, *CES-D* center for epidemiologic studies depression scale, *ECT* Electroconvulsive therapy, *LTL* leukocyte telomere length, *MDD* major depressive disorder, *mtDNAcn* mitochondrial DNA copy number, *TL* telomere length, *TA* Telomere Activity.

**Table 2:** Summary of Telomerase Activity in recent Major Depressive Disorder.

Author, Year	Sample size: MDD /healthy controls	Female (%): MDD/ controls	Duration of study	Attrition rate	Main Findings: TA in MDD	Other findings
Walia, 2023 [33] 10.1016/j.jad.2022.09.138	35 / 35	51.4% / 51.4%	8 weeks	–	Elevated TA in MDD (p = 0.00) TA increases with severity of depression.	Non-responders to antidepressants had significantly higher TA at baseline and post-treatment. Lower baseline TA (pretreatment) showed greater percentage reduction of HAM-D score (i.e. greater response to treatment) (p = 0.010)
Wolkowitz, 2015 [35] 10.1016/j.pscychresns.2015.01.007	Telomerase cohort: 25 /18 LTL cohort: 19 / 17	Telomerase cohort: 68% / 61% LTL cohort: 63%/ 59%	–	–	PBMC TA was significantly higher in the MDD subjects than controls (p = 0.025)	No significant difference in LTL between MDD and controls PBMC TA is significantly and positively correlated with Hippocampal volume in unmedicated individuals with MDD, but not in controls.
Simon, 2015 [6] 10.1016/j.psyneuen.2015.04.004	166 / 166	54%/ 54%	–	–	Significantly higher TA in men with MDD even after adjustment for potential confounders. Higher TA in MDD overall but not statistically significant. No significant difference in TL between MDD and controls.	Depression severity failed to show a significant association with both TL and TA.
Wolkowitz, 2012 [34] 10.1038/mp.2010.133	20 / 18	65%/ 67%	8 weeks	Original depressed cohort n = 20 Treated depressed cohort n = 16 Assessed depressed cohort n = 15	Baseline PBMC TA was significantly elevated in the depressed sample. Elevated resting TA was more apparent in those MDD subjects with poorer treatment response.	Lower baseline (pre-treatment) TA and greater TA increases with antidepressant treatment, showed the greatest benefit from antidepressant treatment. No significant relationships between TA and TL were found.
da Silva, 2022 [5] 10.1097/ypg.0000000000000305	12 / 12	92%	24 weeks	–	After 24 weeks of SSRI treatment and a marked improvement in BDI score, 1. TL significantly increased (P = 0.066) 2. Expression of hTERT significantly increased; TA significantly increased (p < 0.0001)	Shorter TL in MDD group (p < 0.0001)

*BDI* beck depression inventory, *hTERT* human telomerase reverse transcriptase, *TA* telomere activity, *TL* telomere length, *MDD* major depressive disorder, *SSRIs* selective serotonin reuptake inhibitors, *PBMCs* peripheral blood mononuclear cells.

**Table 3:** Summary of Telomere Length in recent Major Depressive Disorder conducted on Brain tissue.

Author, Year	Sample size: MDD /healthy controls	Female (%): MDD/ controls	Tissue source	Duration of study	Attrition rate	Main findings	Other findings
Mamdani, 2015 [14] 10.1038/tp.2015.134	MDD cohort: 10 Controls: 10	MDD cohort: 70% Controls: 30%	Fresh-frozen tissue from 5 brain regions • DPFC • Hip • AmygNA • SN	–	–	Significantly shorter telomeres in the hippocampus of MDDs (1.8-fold reduction)	Most genes forming the telomerase complex (TERC, GAR1, DKC1, NHP2 and NOP10) and the shelterin complex (TPP1, TERF1, TERF2IP, TINF2 and POT1) were expressed at higher levels in the SN when compared with the DPFC, possibly indicating that telomeres are better maintained in theSN.
Szebeni, 2014 [13] 10.1017/s1461145714000698	12 / 12	7.1% / 7.1%	Frozen temporal lobe tissue (containing the UF, and part of the amygdala and hippocampus) or right BA10; Astrocytes and Oligodendrocytes	–	–	Significantly shorter in white matter oligodendrocytes, but not astrocytes.	Lower gene expression of telomerase reverse transcriptase in white matter oligodendrocytes from MDD donors compared to controls. Gene expression of oxidative defence enzymes superoxide dismutase (SOD1 and SOD2), catalase (CAT) and glutathione per- oxidase (GPX1) were significantly lower in oligodendrocytes (not astrocytes) from MDD compared to controls.
Teyssier, 2014 [15] 10.1007/BF03379613	24 /12 MDD with psychosis: 12 Death by suicide: 17	Gender not reported.	DNA of the occipital cortex	–	–	No TL shortening in the cortex of MDD patients, even in long lasting recurring symptoms, psychotic characteristics, or suicidal behaviour	Subjects matched for age, gender, ethnicity, brain PH.

*DPFC* dorsolateral prefrontal cortex, *Hip* hippocampus, *Amyg* amygdala, *NA* Nucleus accumbens, *SN* substantia nigra.

## Alterations of telomere length and telomerase activity in MDD

The majority of studies reviewed reported shorter TL in MDD patients compared to healthy controls, with some studies also noting increased TA in MDD following antidepressant treatment. Among the twenty-two studies in Table 1, eleven found significantly shorter leukocyte TL in individuals with MDD compared to controls [7, 9, 14, 20–27], while four identified a significant association between shortened TL and higher depressive symptom severity [15, 21, 28, 29]. Notably, one study found no baseline TL difference between MDD cases and controls but observed greater TL shortening over a span of two years in MDD cases [30]. This suggests a consistent trend across the majority of studies, indicating an association between MDD and shorter TL. However, notable inconsistencies existed among studies, with seven reporting no significant differences in TL or TA between MDD and control [10, 31–36]. (Tables 1 and 2) Factors such as the age, gender, duration of MDD and comorbidities including metabolic syndrome, cardiovascular and psychiatric disorders (e.g., comorbid anxiety, adverse childhood experiences) influenced TL and TA alterations across these studies [20, 22, 25, 28, 29, 31, 33, 35]. Furthermore, these studies exhibit considerable methodological diversity in terms of setting, study design, sample size, sources of human samples and measurement techniques. They were conducted across various settings, including hospitals, psychiatric clinics, and cohort studies, utilizing both cross-sectional and longitudinal designs. Sample sizes varied from small cohorts with fewer than 100 participants to large-scale longitudinal studies with several thousand participants. The data is based on various sources of telomeres and telomerase activity from human samples (e.g., blood, saliva, brain tissue, cell types, etc.). Brain telomere samples were obtained from post-mortem studies. The studies were primarily cross-sectional with a minority of longitudinal studies. Measurement techniques for TL and TA predominantly included quantitative polymerase chain reaction (qPCR) and quantitative fluorescence in situ hybridization (qFISH), with peripheral blood leukocytes being the most common tissue source.

Results section to describe various sources of telomeres and telomerase activity from human samples (e.g. blood, saliva, brain tissue and cell types, etc), different measures of TL and TA, diagnosis and occurrence of MDD gathered from all the studies for this systematic review.

## Evidence of results

### Telomere attrition in MDD

Several studies which provided evidence supporting the association between depression and telomere attrition in peripheral cells (i.e. leukocytes, peripheral blood monocytes (PBMCs) and salivary cells). Ochi et al. conducted a study among adolescents with MDD, revealing significantly reduced TL and increased mtDNAcn compared to controls, particularly pronounced in those with acute serious suicidal ideation or behaviour. TL shortening also correlated with depression severity, suggesting it as a biomarker for MDD severity. Notably, comorbid anxiety, especially suicidality, also showed significant associations with TL [14].

Da Silva et al. also observed shorter TL in peripheral leukocytes of individuals with MDD, indicative of systemic telomere attrition [9]. Moreover, investigations found that after 24 weeks of selective serotonin reuptake inhibitor (SSRI) treatment and improvement in depression severity, TA significantly increased. This suggests a potential role of psychotropic medication in modulating TA levels, which may influence telomere maintenance [9].

Similarly, Huang et al. also found shorter TL in MDD patients compared to controls. They observed correlations between MDD and socio-demographic and metabolic factors, such as lower education levels, lower employment rates, higher prevalence of cigarette use, and metabolic syndrome (MetS), suggesting an interplay between mental health and various lifestyle and physiological factors contributing to telomere attrition in individuals with MDD. Although the correlation between low high-density lipoprotein cholesterol (HDL-C) levels and shortened TL in MDD cases did not achieve statistical significance after Bonferroni correction, a notable interaction effect between group (MDD vs. controls) and HDL-C levels was observed. MDD patients with low HDL-C levels displayed more pronounced TL attrition compared to controls without low HDL-C levels, implicating oxidative stress as a potential mechanistic pathway [20, 31].

Pisanu et al. revealed a significant reduction in TL in peripheral blood leukocytes. Notably, TL was not significantly correlated with various clinical parameters such as age at onset, years of illness, or number of depressive episodes. Additionally, TL did not differ significantly based on history of suicide attempts or response to antidepressants [7].

In another longitudinal study by Vance et al., subjects with baseline MDD exhibited greater relative TL shortening over two years compared to controls [30]. Additionally, Karabatsiakis et al. found shorter TL in individuals with unipolar depression, persisting independently of symptom severity [23]. Hartmann et al. revealed shorter TL in MDD patients compared to controls, without significant associations with clinical parameters [24].

Furthermore, four studies found significant negative correlation between higher depressive severity and TL shortening. The study by AlAhwal et al. among colorectal cancer patients demonstrated a progressive shortening of TL with increasing severity of depressive symptoms. Higher education level was also associated with shorter telomeres in this cohort [21]. Furthermore, the study by Verhoeven et al. revealed shorter TL in participants with higher depressive symptoms over a 10-year period, with no within-person association between depressive symptoms and TL [15]. Similarly, Whisman et al. found shorter salivary telomeres in men with higher levels of depressive symptoms. The study by Révész et al. also reported shorter TL associated with higher symptom severity and current psychiatric diagnosis [28, 29]. Verhoeven et al. observed shorter TL in both current and remitted MDD patients, with associations with depression severity and symptom duration [22].

### Telomerase activation in MDD

Several studies have reported elevated TA in individuals with MDD compared to healthy controls. Walia et al. found significantly elevated TA in PBMCs of MDD patients, with consistent observations across a longitudinal cohort showing a positive correlation with depression severity [37]. Non-responders to antidepressant treatment exhibited higher baseline TA compared to responders, indicating potential predictive value in treatment response. Similarly, Wolkowitz et al. noted elevated baseline PBMC TA in depressed individuals, particularly in poor responders to antidepressant treatment [38]. Conversely, individuals with lower baseline TA and greater elevations with treatment showed better response, suggesting a predictive role of baseline TA levels in treatment outcomes [38]. Wolkowitz et al. also observed elevated PBMC TA in MDD subjects, positively correlated with hippocampal volume in unmedicated individuals, indicating potential neuroprotective effects [39].

In addition to the above findings, da Silva et al. found that after 24 weeks of SSRI treatment and improvement in depression severity, TA significantly increased, suggesting an influence of psychiatric medication on TA levels [9]. Walia et al. also noted that categorizing patients based on changes in TA and baseline levels revealed that responders tended to have greater treatment-associated changes in addition to lower baseline TA, indicating TA as a potential target for psychopharmacological interventions in MDD [37].

### Reports on negative findings

Eight studies found no significant association between depression and TL or TA. Simon et al. observed higher TA in men with MDD but no significant TL difference [10]. Teysier reported no TL shortening in the cortex of MDD patients, even in those with long lasting recurring symptoms, psychotic characteristics, or suicidal behaviour [40]. Wolkowitz et al. found TL inversely correlated with lifetime depression exposure and oxidative stress, suggesting no intrinsic TL shortening [31]. Ryan et al. found no association between TL and mood ratings or therapeutic response to ECT [32]. Hoen et al. found no association between depressive disorders and TL [33]. Shaffer et al. and Wignall et al. reported no significant TL difference in depression [34, 35]. Needham et al. suggested a potential link between pharmacologically treated MD and shorter TL, highlighting the more severe nature of the condition [36].

## Discussion

Our results yield similar findings to a previous review done in 2015 by Lindqvist et al., which suggested a potential association between TL and PBMC basal TA in full syndromic MDD especially in those with longer illness duration or more severe symptoms albeit with conflicting reports and challenges in drawing definitive conclusions due to small effect sizes, methodological differences and small sample sizes across studies [16].

Since 2015, several new studies have contributed to our understanding of the relationship between TL, TA and MDD. A notable trend observed in these newer studies is the consistent finding of shortened TL in individuals with MDD compared to healthy controls observed in studies by Ochi et al., da Silva et al., Huang et al., Kuehl et al., Pisanu et al., AlAhwal et al., Vance et al., Verhoeven et al., Whisman et al., Révész et al [7, 9, 14, 15, 20, 21, 27–30].

The primary findings align with existing literature, indicating a consistent trend of shortened TL and elevated TA in MDD patients compared to controls, and support our initial hypothesis. Findings such as greater TL shortening over 2 years in MDD patients, shorter TL in those with longer symptoms duration and the inverse association of TL with severity of depression suggest a “dose-response” causal relationship of MDD on TL [15, 21, 22, 28–30]. The absence of significant differences in TL between those with active MDD and those in remission led the researchers to propose that MDD episodes have a lasting impact on LTL. However, they also acknowledged the possibility that TL could be inherently short even before the onset of the first depressive episode, potentially representing a predisposing risk factor for depression [22]. Furthermore, one study has established a connection between hTERT genetic variation and shortened salivary TL in MDD patients, proposing a potential causal role it could play in predisposing individuals to depression [41].

To the extent that TL reflects the effect of cumulative inflammation and oxidative stress exposure, it is possible that longer or more severe depressive symptoms could result in accelerated TL shortening, suggesting a “dose-response” relationship [31]. However, there is also the possibility that TL shortening predates the onset of psychiatric illness or even serves as a risk factor for it, indicating a fixed degree of premature TL shortening regardless of illness severity [16]. Individuals susceptible to MDD, for instance due to family history or adverse childhood experiences, might already have shortened telomeres prior to illness onset and experience further acceleration in telomere shortening with greater exposure to the illness. Although there remains mixed evidences regarding MDD and TL shortening, a relatively consistent finding across studies remains, that both MDD and even anxiety are linked to oxidative stress and inflammation explained by the intense physiological stress experienced [33]. One study showed that anxiety particularly due to its fear and extreme physiological hyperarousal showed more significantly predicted telomere length over time compared to MDD which did not. Further research is needed to uncover the specific mechanisms underlying this bidirectional connection and potentially shed light on the impact of pathophysiologic changes on telomere damage [33].

This underlying mechanism is supported by the fact that effective regulation of emotions can impact a biological marker of stress in individuals with MDD. Higher levels of multisystem resilience, characterized by lower emotion suppression, stronger social connections, increased physical activity, and better sleep quality, were associated with longer telomeres. [42]. Multisystem resilience acted as a moderator between MDD and TL, revealing that individuals with current MDD had significantly shorter telomeres if they had lower resilience, but not if their resilience was higher. This demonstrates how a comprehensive approach to resilience can mitigate the negative effects of depression on cellular aging by disrupting various pathways associated with physiological stress.

However, the evidence for a “dose-response” relationship is suggestive and not entirely consistent. While some studies, such as those by Verhoeven et al. and Wolkowitz et al., found inverse correlations between depression severity or duration and LTL, others, like Hartmann et al., did not observe such relationships [24]. Additionally, findings from Garcia-Rizo et al., who observed decreased telomere content even in first-episode, never-medicated individuals with MDD, raise questions about the timing and progression of telomere shortening in relation to illness onset [26]. The relationship between depression severity and TL is still subjected to much conflicting findings in the literature. Da Silva et al. demonstrated that TL increased following an improvement in depression severity as measured by the BDI score [9]. But, Pisanu et al. found no association between TL and various indicators of depression severity, including illness duration and response to treatment [7]. Two additional studies observed a significant correlation between depression severity and duration, comorbid anxiety, suicidality, and TL [14, 22]. However, seven studies failed to establish a direct link between TL and depression severity [10, 15, 21, 24, 25, 27, 30]. In a similar vein, Karabatsiakis et al. observed no within-person association but hinted at the potential for TL lengthening during remission, thereby suggesting a reversible aspect to telomere dynamics in MDD patients [23].

This review also discovered that MDD subjects with relatively low pre-treatment TA, coupled with greater increases in TA post-treatment, exhibited better responses to antidepressants [37]. This finding suggests that telomerase activation may play a beneficial role in the treatment of depression. This elevated TA in depressed patients may represent an effort to counteract telomere shortening and thereby potentially play a role in mitigating cellular aging processes [38, 39]. However, studies have shown mixed results on the induction of telomerase reverse transcriptase, the catalytic component of telomerase [9, 17]. Considering these findings, telomerase activation is beneficial in depression, and future research on interventions promoting TA may have therapeutic implications for depression and potentially mitigate the adverse effects of telomere shortening.

Our findings also suggest that telomere dysfunction is not an intrinsic factor and does not increase the risk of depression, in line with previous studies by Wolkowitz et al. [31]. However, in our review, somatic comorbidities such as being underweight, overweight or obese, smoking, heavy drinkers, metabolic syndrome, heart disease, diabetes, osteoarthritis, stroke, cancer, etc, were found to be more prevalent in current MDD participants [20, 22, 29]. Prior meta-analysis has suggested an unfavourable impact of somatic comorbidity in MDD on TL and might be a potential mediator of enhanced TL shortening amongst the subset of MDD patients with significant somatic comorbidities [43].

Literature search on TA yielded fewer results compared to TL, indicating that TA in psychiatric illness remains poorly studied. The studies reviewed propose several potential mechanisms underlying the relationship between TA and depression. These include the possibility that TA reflects a compensatory response to cellular damage or to telomere endangerment. Across all TA studies, there’s a consistent positive correlation observed between TA and depression severity [9, 37–39]. Additionally, the findings suggest that telomerase activation could be beneficial in the treatment of depression, although the exact implications of high baseline TA levels are not fully understood. The studies collectively suggest that baseline TA levels can predict the response to antidepressant treatment. Specifically, individuals with lower baseline TA tend to exhibit a better response to antidepressants, while those with higher baseline TA may respond less effectively or even poorly to treatment [37, 38]. Additionally, greater increases in TA during antidepressant treatment are associated with better treatment response [9, 38].

Regardless, the underlying mechanisms for the observed associations between TA and depression still remain uncertain as it may indicate compensatory mechanisms in response to shorter telomeres or that higher baseline TA could indicate more underlying depression-related pathology, potentially induced by greater telomerase activation [37, 38]. The limited number of studies, inconsistent findings, and variations in approach (e.g. duration and type of antidepressants) make it challenging to arrive at a definitive conclusion. Nevertheless, it’s plausible that TA and TERT are influenced by stress and play a role in the progression of MDD and response to antidepressants.

Despite evidence supporting the connection between depression and telomere attrition, there exist conflicting findings and gaps in our current understanding. Notably, while some studies report shorter TL in MDD patients, others fail to find significant differences compared to healthy controls as a whole [10, 27, 30–36, 39]. Such inconsistencies may stem from variations in sample characteristics (e.g. sample size, gender, age, existing comorbidities), measurement techniques, and the influence of confounding factors, warranting further exploration. For instance, AlAhwal et al. found that higher education levels were associated with shorter telomeres, possibly due to increased stress from greater responsibilities and knowledge of disease consequences [21]. Conversely, Karabatsiakis et al. did not assess various factors such as body mass index, lifestyle habits, or cardiovascular health, potentially impacting TL [23]. Hartmann et al. did not control for factors like gender, race, smoking, or socio-economic status, with all participants being Caucasian inpatients, limiting generalizability [24]. Hoen et al. suggested that TL differences between depression groups might be confounded by cardiac disease severity, while Garcia-Rizo et al. had a small MDD sample size with limited information on participant characteristics [25, 26]. Whisman et al. observed that their study’s age restriction might affect TL variability, and their measures of traumatic life events and neuroticism were brief [28].

As shown in the tables above, many studies had a slightly higher percentage of female participants. Although majority of studies supporting our hypothesis had already controlled for age and sex, there are still mixed results with some showing a potential age- or sex-moderated association between TL and MDD. For example, in the study by Whisman et al., after adjusting for demographic variables, sex moderated the association between depressive symptoms and salivary TL, such that it was statistically significant for men but not for women [28]. Additionally, Verhoeven et al. saw an age- and sex-moderated association with LTL [15].

### Other biomarkers of cellular aging in MDD

Studies have shown mixed results regarding the association between mitochondrial DNA (mtDNA) biomarkers and MDD. Lindqvist et al. found elevated levels of cell-free mitochondrial DNA (ccf-mtDNA) in individuals with MDD, particularly in non-responders to SSRI treatment, while Verhoeven et al. did not find a between- nor within-person association between depressive symptoms and mtDNA copy number (mtDNAcn) over a 10-year follow-up period [15, 44]. Ochi et al. reported reduced telomere length (TL) and increased mtDNAcn in adolescents with MDD, especially in those with acute suicidal ideation or behavior [14]. These discrepancies may stem from differences in study design, sample characteristics, and measurement techniques, underscoring the complexity of the relationship between mtDNA and MDD. Two studies from Ochi et al. [14] and Verhoeven et al. [11] are from the same studies presented in this systematic review (Table 1).

### Telomere length as a diagnostic tool or an effect of MDD?

The evidence presented in the reviewed studies suggests that TL alterations may serve as both potential diagnostic indicators and consequences of MDD. Several studies reviewed here provide support for the notion that shortened TL may serve as a potential biomarker or consequence of MDD [9, 44]. Notably, many studies consistently reported shorter TL in individuals with MDD compared to healthy controls, suggesting a potential role of TL in reflecting the biological processes underlying MDD pathophysiology. Moreover, alterations in TA observed in MDD patients, particularly in response to antidepressant treatment, further support the involvement of telomere dynamics in the disorder’s progression and treatment response [37, 38]. Wolkowitz et al. concluded that telomere shortening is an effect of MDD rather than a predisposing factor [31]. However, conflicting findings and methodological inconsistencies complicate the interpretation of TL alterations in MDD, suggesting a more complex relationship between TL and MDD [14, 15]. While TL alterations may reflect underlying biological processes in MDD, such as oxidative stress and inflammation, the lack of consistent associations between TL, depression severity, certain socio-demographic and metabolic factors, suggests a more intricate relationship that warrants further investigation [7, 20, 23].

### Is cellular aging in MDD reversible?

There is suggestive evidence that aspects of cellular aging, particularly TL dynamics, may be reversible under certain conditions, such as during remission from MDD and with the use of antidepressants, thereby elevating TA.Studies conducted by da Silva et al. observed an increase in TA following 24 weeks of SSRI treatment and improvement in depression severity, indicating a potential role of psychotropic medication in modulating TA levels, and potentially influencing TL maintenance [9]. This suggests that effective treatment of MDD may lead to improvements in TL dynamics, possibly reversing telomere attrition associated with the disorder. Similarly, Karabatsiakis et al. hinted at the potential for TL lengthening during remission from depression, suggesting a reversible aspect to telomere dynamics in MDD patients [23].Additionally, Mamdani et al. reported elevated TA in PBMCs in individuals with MDD, which was positively correlated with hippocampal volume. This suggests a potential protective mechanism against cellular aging in MDD, hinting at a compensatory response that may contribute to TL maintenance or even lengthening in certain contexts [18].

However, it’s important to note that the evidence for TL lengthening in remission is not definitive and is based on a limited number of studies. Further research is needed to fully understand the reversibility of TL alterations in MDD and to identify the specific factors and mechanisms involved in TL dynamics during remission. Additionally, other aspects of cellular aging, such as mitochondrial DNA dynamics, may also play a role in the reversibility of cellular aging processes in MDD, although more research is needed in this area as well [44].

### Studies conducted on brain cells

Three studies (Table 3) examining human brain cells shed light on the intricate relationship between MDD and telomere/telomerase dynamics. Szebeni et al.‘s discovery of shorter telomeres in white matter oligodendrocytes of depressed individuals suggests that cellular ageing processes, at least within certain brain cell types, may contribute to the pathophysiology of depression [17]. Oligodendrocytes are important for myelination of the neuron and structural integrity of the brain’s white matter, and this observed reduction in telomerase gene expression and protective oxidative enzymes further implicates impaired cellular maintenance mechanisms in depression-related cellular ageing.Mamdani et al. conducted a quantitative survey of TL in five brain regions of psychiatric patients, including those with MDD, bipolar disorder (BD), and schizophrenia (SZ), along with normal controls. They observed a significant 1.8-fold reduction in TL specifically in the hippocampus of individuals with MDD, linking telomere length reductions in the human hippocampus to MDD. The study also noted variable TL across different brain regions and revealed significantly decreased expression of GPR37 and HSPA2 genes, along with higher expression levels of PPARD and PPARG genes in MDD subjects. The study suggests varying rates of cellular aging across brain regions, with a pronounced increase in the hippocampus of MDD subjects, consistent with previous findings of hippocampal volume reduction in MDD [18, 45]. However, older studies like Teyssier et al. present a contradictory result, failing to find telomere shortening in cells from the occipital cortex of depressed patients [40]. Authors also suggest an unlikely impact of peripheral oxidative states measured by peripheral gene expression (OGG1 gene) on cerebral pathological changes. This discrepancy raises questions about the regional specificity of telomere dynamics within the brain and emphasises the need for further research to elucidate the intricacies of cellular ageing processes in different brain regions. This also suggests that telomere dynamics within central neurons may not always follow the same patterns observed in peripheral cells or other brain cell types [40]. From these findings, the hippocampus appears most vulnerable to stress, with hippocampal TL and TA potentially playing a role in neuronal functioning, structural changes, and the onset of premature aging associated with depression. Additionally, there is intriguing evidence suggesting that TL in oligodendrocytes is affected by depression. While it was anticipated that TL or TA might be impaired in microglia cells or astrocytes, recent preclinical and clinical studies have instead pointed towards impaired interactions between neurons and glial cells, as well as disruptions in plasticity, as potential key mechanisms underlying depression [46].

## Conclusion

Overall, these studies collectively suggest a nuanced relationship between depression and telomere/telomerase dynamics, with potential implications for both the aetiology and treatment of depression. Further investigation into the mechanisms underlying these associations may offer novel insights into the development of targeted interventions aimed at mitigating cellular ageing processes. It’s possible that cellular aging processes in depression involve complex interactions between various cell types and systems throughout the body, both peripheral and central.
